# Study on wastewater toxicity using ToxTrak™ method

**DOI:** 10.1007/s11356-016-6096-4

**Published:** 2016-02-01

**Authors:** Ewa Liwarska-Bizukojc, Radoslaw Ślęzak, Małgorzata Klink

**Affiliations:** Institute of Fermentation Technology and Microbiology, Lodz University of Technology, Wolczanska 171/173, 90-924 Lodz, Poland; Department of Bioprocess Engineering, Lodz University of Technology, Wolczanska 213, 90-924 Lodz, Poland; Institute of Environmental Engineering and Building Installations, Lodz University of Technology, Al. Politechniki 6, 90-924 Lodz, Poland; Water Supply System and Sewer–Zgierz Ltd., ul. A. Struga 45, 95-100 Zgierz, Poland

**Keywords:** Activated sludge, Monitoring, Toxicity, ToxTrak™ method, Validation, Wastewater

## Abstract

ToxTrak™ method is an analytical tool for the measurement of toxicity of drinking water, wastewater and natural water. It is based upon the estimation of the inhibitive effect on bacterial respiration processes. The main aim of this work was to test the applicability of ToxTrak™ method in the assessment of wastewater toxicity in a full-scale WWTP in Poland. In order to achieve it, the study was divided into two parts. First, the validation of ToxTrak™ method was performed. Second, wastewater toxicity was monitored in the long- and short-term campaigns. Validation of ToxTrak™ method revealed that the indigenous biomass (mixed cultures of activated sludge microorganisms) was more sensitive than *Escherichia coli* for both materials (wastewater and phenol) tested. The values of degree of inhibition determined for phenol towards indigenous biomass and *E. coli* were close to each other, and no statistically significant difference between them was found. It confirmed the reliability of the results obtained with the help of ToxTrak™ test. The toxicity of the effluent was always lower than that of the influent and the linear correlation between them was found. Despite, the decrease of wastewater toxicity in the WWTP, the effluents were ranked as toxic or highly toxic according to the classification of wastewater based upon the acute toxicity.

## Introduction

In the last decade, not only the removal of macropollutants but also that of micropollutants from wastewater was intensively investigated. Wastewater is a complicated matrix of compounds, some of them are present in very small amounts (below 1 mg l) and, what is more important, their presence does not influence on the values of such combined indicators of contamination as chemical oxygen demand (COD). It suggests that the standard physicochemical determinations used for the characterisation of wastewater composition are not sufficient nowadays. Modern chromatographic methods coupled to mass spectrometry allow for the determination of the individual components of wastewater (i.e. selected pharmaceuticals, pesticides or detergents); however, they do not quantify the effect of wastewater on living organisms and do not assess the environmental risk. For this purpose, the application of toxicity tests is desirable. It is regarded that the evaluation of toxicity is necessary to complement the physicochemical measures of wastewater quality (Hernando et al. 2005; Libralato et al. 2010a). Such holistic attempt is particularly important in the countries facing water scarcity, in which water reuse is widely implemented.

A variety of tests were used over the last years in order to estimate the toxicity of wastewater. The most common were bioluminescence tests with *Vibrio fischeri* and growth inhibition tests with *Pseudomonas putida*, the acute immobilization tests with *Daphnia magna* and algae growth inhibition tests (Hernando et al. 2005; Ra et al. 2007; Libralato et al. 2010b; Vasquez and Fatta-Kassinos 2013; Tobajas et al. 2015). Also, other organisms like diatom *Phaeodactylum tricornutum* Bohlin (Libralato et al. 2016) and bivalve molluscs (Libralato et al. 2010a) were applied in order to evaluate wastewater toxicity and/or efficiency of wastewater treatment processes. It shows that wastewater toxicity was measured towards pure cultures of organisms representing different trophic levels, i.e. producers, consumers and decomposers. These tests were usually made in agreement with the OECD standard procedures or the ISO norms.

In parallel, several classifications of wastewater toxicity were developed. They were described in detail elsewhere (Libralato et al. 2010b). One of the most commonly used is the classification proposed by Persoone et al. (2003), which distinguished five classes of wastewater toxicity dependent on the value of toxicity units (TU).

Literature review revealed that there was a lack of data concerning wastewater toxicity towards mixed cultures of organisms, while the mixed cultures are exposed to wastewater in biological treatment processes. Also the number of scientific papers presenting the variations of wastewater toxicity in the long-term period and/or seeking for the correlation between the physicochemical measures and toxicity is very limited (Ra et al. 2007; Yi et al. 2009; Vasquez and Fatta-Kassinos 2013; Xiao et al. 2015).

In this work, ToxTrak™ toxicity test using the indigenous biomass (mixed cultures) was a tool for the measurement of wastewater toxicity. The main aim of the study was to test the applicability of ToxTrak™ method in the assessment of wastewater toxicity in a full-scale WWTP.

In order to achieve this aim, the work was divided into two parts. First, the validation study for ToxTrak™ method was performed. Second, for better assessment of wastewater toxicity, its variability was monitored in the long-term and short-term campaigns. The second part of this work was also aimed at the evaluation of toxicity reduction and seeking for the correlations between physicochemical measures of wastewater composition and its toxicity.

## Materials and methods

### Description of the WWTP

**Fig. 1:**
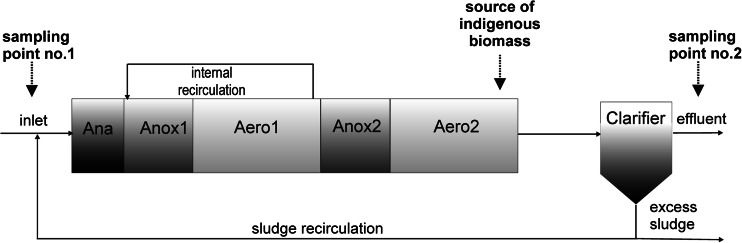
Scheme of the activated sludge system of the WWTP Zgierz including the sampling points

The object of this study was the WWTP Zgierz (Poland) that generally treats municipal wastewater. The contribution of industrial wastewater usually varies from 7 to 15%. The average pollutant load to the plant corresponds to approximately 94,000 PE. The biological stage consists of one five-zone bioreactor and secondary clarifier run in the Phoredox process configuration. The total volume of bioreactor is 24,000 m^3^. The scheme of the biological system working in the WWTP Zgierz including the sampling points is depicted in Fig. 1. Hydraulic retention time of wastewater in the WWTP studied was about 48 h; thus, the effluent (sampling point no. 2) was each time sampled with 2-day delay in order to relate it to the influent properly. The sampling dates presented in the figures and tables correspond with the date of the sampling of influent.

In the period of study, i.e. from August 2013 to August 2014, the average inflow of wastewater was 8725 m^3^ day^−1^, reaching the maximum value of 17,300 m^3^ days^−1^ in October 2013. BOD_5_ of the influent varied from 425 to 6520 mg O_2_ l^−1^, while COD was in the range from 632 to 8020 mg O_2_ l^−1^. BOD_5_/COD ratio varied from 0.202 to 0.696. The concentration of total nitrogen in the influent was in the range from 67.8 to 98.3 mg l^−1^, while the total phosphorus varied from 8.9 to 22.4 mg l^−1^. More detailed characteristics of the influent is presented in Table 1. In the period of study, the levels of carbon, nitrogen and phosphorus removal from wastewater required by Polish legislation were achieved in this plant. There were no significant disturbances in the operation of the WWTP under study.

**Table 1:** Characteristics of the influent and effluent in the WWTP studied

Characteristic feature	Short-term campaign		Short-term campaign		Long-term campaign	
	January 2014		June–July 2014			
	Influent (sampling point no. 1)	Effluent (sampling point no. 2)	Influent (sampling point no. 1)	Effluent (sampling point no. 2)	Influent (sampling point no. 1)	Effluent (sampling point no. 2)
pH (−)	7.0–7.7	7.0–7.3	7.3–7.6	7.1–7.2	7.0––7.8	7.0–7.3
COD (mg O_2_ l^−1^)	632–3932	34–59	1240–3760	24–84	632–8020	19–84
BOD_5_ (mg O_2_ l^−1^)	440–1200	2–10	425–980	2–11	425–6520	2–12
Conductivity (μS cm^−1^)	1587–2740	1502–2220	1748–2420	1694–1946	1505–2740	1461–2220
Ammonium (mg N-NH_4_^+^ l^−1^)	57.63–79.59	0.42–0.65	66.92–70.21	0.29–0.79	57.53–79.59	0.28–0.79
N_tot_ (mg N l^−1^)	67.81–98.26	5.06–5.71	75.19–84.59	3.94–6.14	65.38–98.26	5.06–7.42
P_tot_ (mg P l^−1^)	8.9–22.4	0.21–0.40	9.5–14.4	0.30–0.69	8.9–23.3	0.21–0.69

### ToxTrak™ test

ToxTrak™ method was elaborated to be an analytical tool for the measurement of toxicity of drinking water, wastewater and natural water. The method is based on the reduction of resazurin, a redox-active dye, by bacterial respiration. When it is reduced, resazurin changes its colour from blue to pink. The presence of toxic substances in the sample decreases the rate of resazurin reduction, which can be measured colorimetrically. The endpoint of ToxTrak™ method is the inhibition of bacterial respirometric activity. The results of this test (toxicity scores) were expressed as the degree of inhibition (DI) in a percentage (%). In order to make them more universal and easier comparable to other toxicity data, the values of inhibition concentration (IC50) were determined by the linear regression between wastewater concentration (diluted samples of wastewater) and the degree of inhibition in a logarithmic coordinates system. Then, the toxicity units (TU) were calculated (Swedish EPA 1997; Libralato et al. 2010a; Vasquez and Fatta-Kassinos 2013).

In this work, ToxTrak™ test was used to measure the toxicity of municipal wastewater from the WWTP Zgierz (Poland). Inoculum was basically the indigenous biomass taken from the second aeration chamber of the Zgierz WWTP (Fig. 1) and prepared in agreement with the guidelines delivered by HACH Company. Absorbance was measured with the use of spectrophotometer DR 6000 at *λ* = 603 nm. The test was made in accordance with the guidelines for ToxTrak™ (Toxicity ToxTrak™ Method 10017, HACH LANGE Manual. http://www.hach.com/toxtrak-toxicity-reagent-set-25-49-tests/product-downloads?id=7640273469). Each sample was made in five replications. If necessary, additional replications were made in order to obtain reliable results.

### Physicochemical analyses

Apart from toxicity, the physicochemical indicators of wastewater (influent and effluent) were also determined in agreement with the standard methods. These were pH, COD, BOD_5_, conductivity, ammonium and total nitrogen and total phosphorus (APHA-AWWA-WEF 2012). Their values for the influent are included in Table 1.

### Validation study

**Fig. 2:**
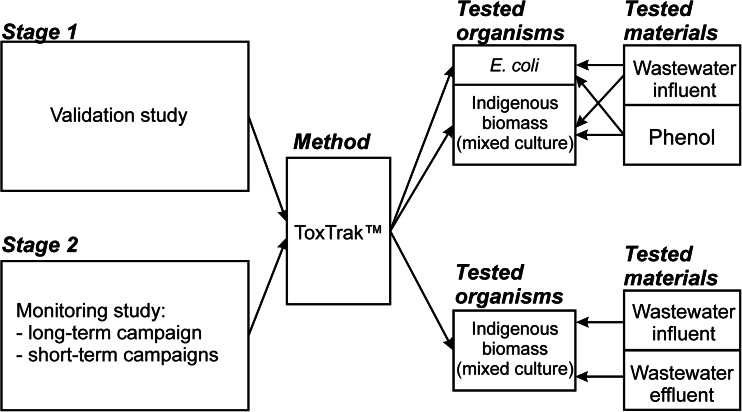
Experimental scheme for the wastewater toxicity study

The application of any toxicity test in new conditions or for a new object requires the validation of the experimental procedure to evaluate its sensitivity and precision. Here, the validation of ToxTrak™ method comprised two stages (Fig. 2). First, ToxTrak™ test was conducted with two different inocula, i.e. the indigenous biomass (mixed culture) from the WWTP Zgierz (Fig. 1) and pure culture of *Escherichia coli* from the collection DSM 30083 (Fig. 2). Activated sludge used in the tests was characterised by high biodiversity and its biotic index varied from 6 to 8 (Madoni 1994). Second, two types of tested materials were used. These were raw wastewater (influent) from the WWTP Zgierz and the solution of phenol. Phenol is recommended as the organic reference substance for testing of toxicity towards bacteria (Nalecz-Jawecki et al. 2010; Microtox® Acute Toxicity Test 1998).

### Monitoring of wastewater toxicity

The monitoring study was performed from August 2013 to August 2014 in the WWTP Zgierz (Fig. 2). Toxicity of raw wastewater (influent) and treated wastewater (effluent) was determined (Fig. 1). The averaged diurnal samples of the influent and effluent were taken from the WWTP Zgierz under dry weather conditions and tested within 2 h.

Two types of the measurement campaigns, i.e. long and short term, were conducted. In the long-term campaign, toxicity was measured once a month over 13 months. During the short-term campaigns, the samples were taken three times a week. Each short-term campaign lasted 15 days and comprised seven samples. One short-term campaign was conducted in winter (January 2014) and the other one in summer (June and July 2014).

### Data analysis

**Fig. 3:**
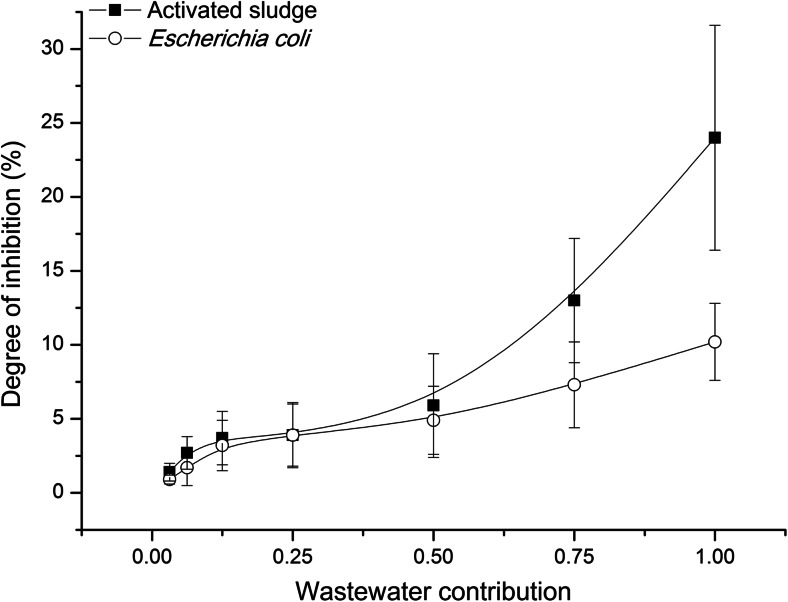
Wastewater toxicity of towards activated sludge microorganisms and *Escherichia coli*

**Fig. 4:**
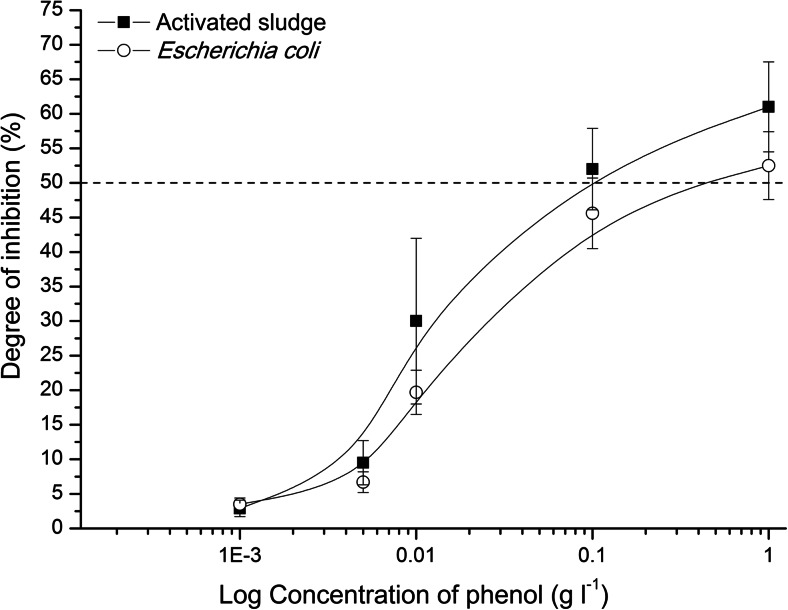
Toxicity of phenol towards activated sludge and *Escherichia coli*

**Fig. 5:**
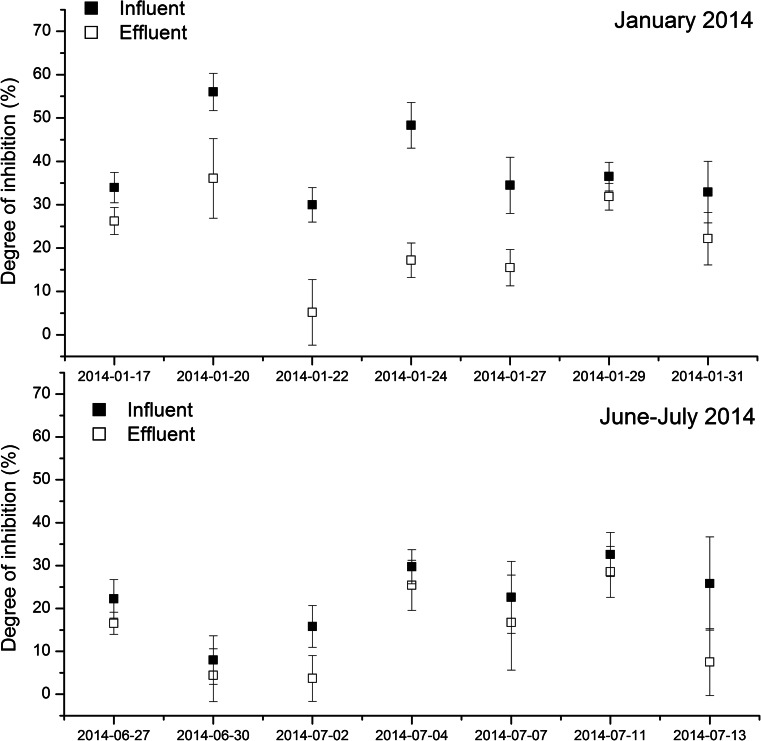
Variations of toxicity of raw and treated wastewater during the short-term campaigns

**Fig. 6:**
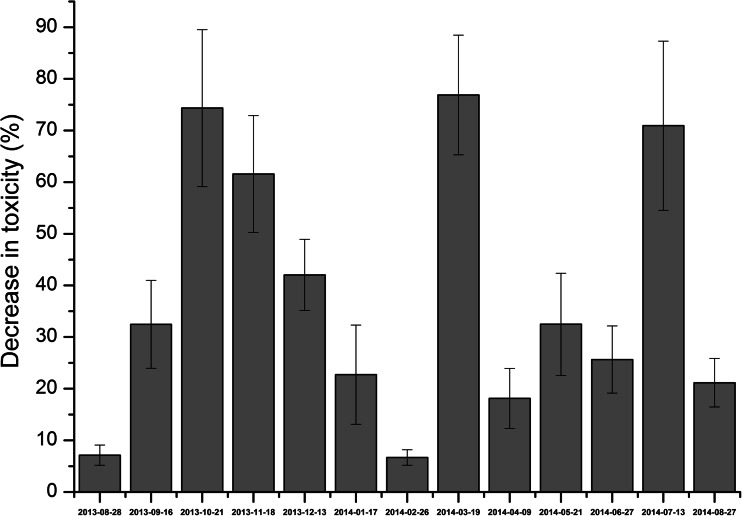
Variations of the decrease of wastewater toxicity during the long-term campaign

Toxicity score in ToxTrak™ method was expressed as the percent inhibition degree. In accordance with the guidelines of the test (Method 10017, HACH, Loveland, CO, USA), each sample was tested in five replications. The results of tests were subjected to the basic statistical analysis that comprised the calculation of mean values, standard deviations and relative standard deviations (RSD) of the measured degrees of inhibition. Linear regression (*R*^2^) and Pearson’s coefficients were used in order to find the correlation between toxicity and physicochemical indicators of wastewater or operation parameters of the WWTP studied. Moreover, linear regression was applied to seek for the correlation between influent and effluent toxicity. Both basic statistical analysis and correlation analysis were made with the use of MS Excel. The confidence level of 95 % was each time assumed. Results (from Figs. 3, 4, 5 and 6) were presented as mean values with the standard deviations.

Additionally, one-way analysis of variance (ANOVA) was applied to evaluate whether the degrees of inhibition in the validation study were equal. The null hypothesis was that they were equal. The confidence level of 95 % was assumed, too. ANOVA implemented in MS Excel (Analysis ToolPak) software was used.

## Results and discussion

### Validation study

In the validation study, the toxicity of raw wastewater from the WWTP Zgierz towards mixed culture (activated sludge microorganisms) and pure culture (*E. coli*) was tested. It was also made for phenol solutions (Fig. 2). The results of these tests are presented in Figs. 3 and 4, respectively. The curves illustrating the changes of the degree of inhibition dependent on wastewater dilution or phenol concentration had the same shape for both types of biomass (mixed and pure culture) used. The values of the degree of inhibition determined for activated sludge and *E. coli* were often close to each other. Taking the fact that the ranges of standard deviations calculated for the degree of inhibition towards mixed and pure culture covered themselves into account, it could be claimed there was no statistically significant difference between them. It concerned the values of the degree of inhibition estimated for phenol and for more diluted wastewater (wastewater contribution up to 0.5 *v*/*v*). The exceptions were undiluted or slightly diluted (wastewater contribution 0.75 *v*/*v*) raw wastewater (Fig. 3), when the discrepancy between the degree of inhibition for activated sludge and the one for *E. coli* reached even 200 %. The most probable reason was the material tested, i.e. wastewater, which was the complicated matrix of compounds. In the tests with phenol, such discrepancies between the degrees of inhibition calculated in the tests towards activated sludge and *E. coli* were not observed (Fig. 4). The results of one-way ANOVA test confirmed that there was no sufficient evidence to reject the null hypothesis that the group means were equal (*P* value >*α*, where *α* = 0.05) with regard to both experiments, i.e. the tests with phenol (*P* value = 0.334) and the tests with wastewater (*P* value = 0.673).

The reproducibility of ToxTrak™ tests, calculated as the relative standard deviation (RSD), varied from 9.3 to 52.6%. The lowest values up to 24.2% were found in the tests, in which toxicity of phenol towards *E. coli* was measured. They were in agreement with the results obtained by Hernando et al. (2005), where RSD was in the range from 5 to 22.3% in the toxicity tests with the use of *Vibrio fisheri*, *Selenastrum capricornotum* and *D. magna*. The application of activated sludge biomass in place of *E. coli* in the experiments with phenol caused to the decrease of reproducibility of the ToxTrak™ test (higher RSD up to 39%). It was the most probable associated with the biodiversity of activated sludge biomass used in the tests. Nevertheless, the application of ToxTrak™ test using indigenous biomass in a WWTP can help to recognise the impact of influent on the biomass, which is responsible for the microbiological removal of pollutants from wastewater. As a result, the operators can avoid disturbances in functioning of the biological part of a WWTP.

In ToxTrak™ tests, the indigenous biomass as well as pure culture can be used as the inoculum. The comparison of these two types of inocula made here showed that activated sludge microorganisms were more sensitive than *E. coli*, irrespective of the tested material (wastewater or phenol solution) (Figs. 3 and 4). The agreement between these results confirmed the correctness of the laboratory work performed. The inhibition concentration IC50-45 min estimated for phenol was 49.3 mg l^−1^ towards activated sludge biomass and 373 mg l^−1^ in the tests with *E. coli*. The value of IC50-5 min for phenol towards *V. fischeri* in Microtox® Acute Toxicity Test should be included in the range from 13 to 26 mg l^−1^ (Modern Water Microtox Acute Toxicity and Modern Water 1998).

Lower value of IC50-45 min determined in the tests with the use of indigenous biomass compared to these with *E. coli* indicated on the higher resistance of *E. coli* towards tested materials (wastewater or phenol solution)*.* Vasquez and Fatta-Kassinos (2013) found that wastewater samples were less toxic to *V. fischeri* than *Pseudokirchneriella subcapitata* or *D. magna*. But it must be remembered that activated sludge is a mixture of organisms consisting mainly of bacteria and protozoa and its sensitivity may vary to a higher extent than it may in the case of pure cultures.

Basically, the organisms used in the toxicity tests should be sensitive to a variety of chemicals (U.S. EPA 2002). Thus, activated sludge organisms were selected as the inoculum for the further tests made within the monitoring study. The second reason of this choice was the fact that these organisms were exposed to raw wastewater in the biological step of the WWTP and the results of toxicity tests allowed for the prediction, whether the influent could exert any effect on biological treatment processes. As a result, the relationships between toxicity of raw wastewater and the efficiency in removal of C, N and P from wastewater could be found.

### Monitoring study

The toxicity of raw wastewater expressed as the degree of inhibition varied significantly from 10.2 to 59.8%, whereas for the treated ones from 3.3 to 35.6%. Such variability of results concerned also other measures as COD or BOD_5_ and is typical for wastewater being the mixture of various compounds. What is more, the composition of this mixture varied in time. The variations in wastewater toxicity were observed in the long- as well as in the short-term campaigns. Yi et al. (2009) and Vasquez and Fatta-Kassinos (2013) found the great changes in the toxicity of wastewater in the monitoring study of the WWTPs, too. Furthermore, Vasquez and Fatta-Kassinos (2013) observed significant variations of toxicity dependent on the season and the species tested.

In this work, variations of wastewater toxicity in respect to the season were also apparent. The results of the short-term campaigns revealed that raw wastewater were more toxic in winter than in summer (Fig. 5). The mean value of DI in the whole winter campaign was equal to 38.9 %, while in the summer one, it was 22.4 %. The reason was the most probably connected with the activity of small businesses including factories, from which wastewater was delivered by sewer system to the studied WWTP. Their activity was higher in January in comparison to the end of June and July, when many of them reduced or even stopped their manufacturing due to the holidays. It was confirmed by the contribution of industrial wastewater in the influent, which was at the level of 12 % in the winter campaign and 7 % in the summer one. Ra et al. (2007) observed higher toxicity of municipal wastewater in winter than in summer, too. At the same time, Vasquez and Fatta-Kassinos (2013) found the opposite relation. According to their work, municipal wastewater toxicity was higher in summer than in winter due to lower dilution of wastewater in the summer period. However, it should be added that in the study performed by Vasquez and Fatta-Kassinos (2013), four samples per year were taken, whereas in this work, each short-term campaign comprised seven samples. It makes the results presented here more reliable with regard to the seasonal variations of toxicity in the WWTP Zgierz.

Irrespective of the sampling date, the toxicity of the influent was higher than the toxicity of the effluent (Fig. 5). It indicated that the biological treatment system reduced wastewater toxicity. However, in some cases, the difference between the toxicity of raw and treated wastewater was relatively small. The decrease of toxicity in the WWTP studied varied widely from 5.8 to 76.6% (Fig. 6). There were several possible reasons of the variability of these results. The most probable was the composition of raw wastewater and operational parameters of the activated sludge system. Yi et al. (2009) also observed the decrease of wastewater toxicity after biological step and secondary clarifier; however, after Fenton process, the toxicity increased again. There was no correlation between the decrease of toxicity and the biodegradability of the influent expressed as BOD_5_/COD (*R*^*2*^ below 0.200). Also, such operational parameters as sludge loading rate, sludge age or temperature in the bioreactor did not have any unequivocal effect on the decrease of wastewater toxicity (*R*^2^ below 0.200). Thus, it is difficult to say, which operational conditions favour the decrease of wastewater toxicity.

**Fig. 7:**
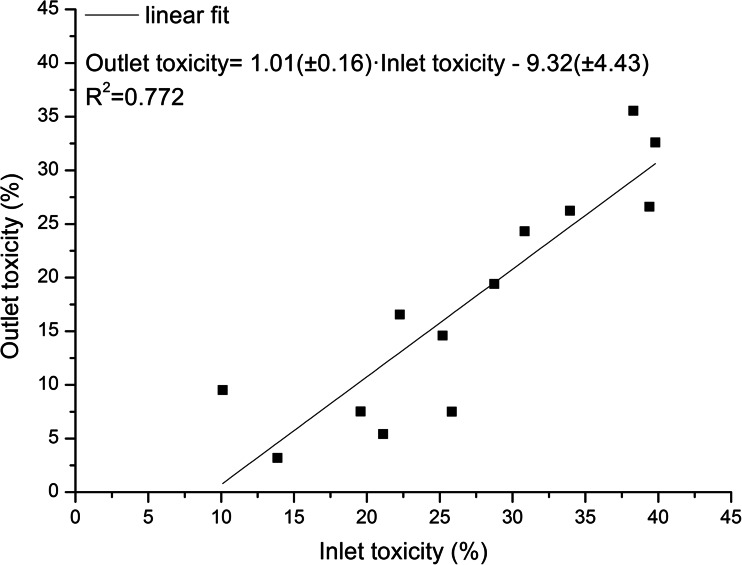
Correlation between toxicity of influent and effluent

At the same time, the correlation between the toxicity of the influent and effluent was found. The coefficient of determination (*R*^2^) was equal to 0.772 (Fig. 7). Generally, the higher inlet toxicity is, the higher toxicity of the effluent can be expected.

Additionally, it was checked whether the toxicity of influent was correlated with physicochemical indicators of raw wastewater or degree of removal of organic compounds (COD), nitrogen or phosphorus. The values of Pearson’s coefficient and *R*-squared are presented in Table 2. Unfortunately, the obtained results did not allow for the statement that the toxicity of raw wastewater was correlated with any of physicochemical indicator or biodegradability. The values of *R*-squared were usually below 0.200 (Pearson’s coefficients below 0.400). According to Postma et al. (2002) ammonium and conductivity were considered as “confounding factors” that may interfere with the biological effects of micropollutants removal. It was found elsewhere that the conductivity of raw wastewater was correlated with the toxicity to *D. magna* (Vasquez and Fatta-Kassinos 2013). Therefore, the correlations between toxicity and these two parameters were checked carefully. Only a weak correlation was found between the conductivity and toxicity of raw wastewater in the summer campaign (*R*^2^ = 0.521) (Table 2). At the same time in winter and long-term campaigns, such correlation was not observed (*R*^2^ below 0.100) (Table 2). Toxicity of raw wastewater, if it was in the measured range (DI from 10.2 to 59.8 %), did not interfere with the efficiency of organic carbon (COD), nitrogen and phosphorus removal. As written earlier, the levels of carbon, nitrogen and phosphorus removal from wastewater required by Polish legislation were achieved in the WWTP studied in each measurement campaign.

**Table 2:** Pearson’s and *R*-squared coefficients for the correlations between toxicity and physicochemical indicators of raw wastewater (influent) and between toxicity of raw wastewater and efficiency of removal of organic compounds and nutrients

Correlation	Pearson’s coefficient/*R* ^2^		
	Short-term campaign	Short-term campaign	Long-term campaign
	January 2014	June–July 2014	
Toxicity vs. COD	0.231/0.0534	−0.388/0.151	0.0701/4.92 · 10^−3^
Toxicity vs. BOD_5_/COD	0.171/0.0292	0.371/0.138	0.138/0.0191
Toxicity vs. conductivity	0.0537/2.88 · 10^−3^	0.721/0.521	0.231/0.0534
Toxicity vs. ammonium concentration	0.189/0.0357	−0.177/0.0313	0.301/0.0906
Toxicity vs. removal of COD	0.191/0.0365	0.358/0.128	−0.0592/3.50 · 10^−3^
Toxicity vs. removal of nitrogen	−0.403/0.162	−0.0359/1.29 · 10^−3^	−0.238/0.0566
Toxicity vs. removal of phosphorus	0.405/0.164	−0.389/0.151	0.454/0.206

What is more, no strong linear correlation between toxicity of raw wastewater and degree of removal of COD, N_tot_ and P_tot_ was found. The values of *R*-squared and Pearson’s coefficients were from 1.29 · 10^−3^ to 0.206 and from −0.403 to 0.454, respectively (Table 2). A weak or moderate positive correlation was found between toxicity of wastewater and phosphorus removal (Table 2). A weak negative correlation was observed between toxicity of wastewater and nitrogen removal too but it concerned the winter campaign only (Table 2).

Although the toxicity of effluent was always lower than that of influent, it did not mean that the treated wastewater was environment friendly. According to the classification of wastewater toxicity based upon the acute toxicity proposed by Persoone et al. (2003), the effluent was toxic (class III) or highly toxic (class IV). Out of 25 samples of the treated wastewater, two belonged to class III and 23 samples were ranked as class IV. The results of toxicity tests performed to *V. fischeri* indicated that the effluent of the WWTP tested by Vasquez and Fatta-Kassinos (2013) was usually classified as class III or IV, i.e. similarly as in this work. Toxic properties of the treated wastewater meant that its introduction to water bodies could adversely affect the organisms living there as well as functioning of the whole ecosystem. Furthermore, it may have the influence on public health. In order to decrease the risk of water toxicity and to protect water environment, the additional treatment after biological step should be implemented in wastewater treatment plants, particularly in the countries facing water scarcity problems.

## Conclusions

The validation study revealed the usefulness of Toxtrak™ test in the measurement of wastewater toxicity. Both indigenous biomass from wastewater treatment plants and *E. coli* can be successfully applied in the measurement of wastewater toxicity. Indigenous biomass was more sensitive than *E. coli* for both materials (wastewater and phenol solution) tested. The values of the degree of inhibition determined for phenol solutions towards activated sludge microorganisms and *E. coli* were close to each other and no statistically significant difference between them was found. It confirmed the reliability of the results obtained with the help of Toxtrak™ test.

The toxicity of wastewater in the short- as well as long-term campaigns varied widely. In respect to the season, lower toxicity of raw wastewater was observed in summer than in winter. The toxicity of the effluent was always lower in comparison to the influent. The linear correlation between the toxicity of the influent and effluent was found. At the same time, any strong linear correlation between the toxicity of raw wastewater and physicochemical parameters (pH, COD, ammonium nitrogen, total nitrogen, total phosphorus) or biodegradability (BOD_5_/COD) was not observed. Despite the fact that the WWTP effluents obeyed all regulations in Poland with respect to the physicochemical properties, they were not biologically safe. They were ranked as toxic or highly toxic according to one of the classifications of wastewater based upon the acute toxicity.

This work confirms that apart from traditional, physicochemical measures of wastewater composition, also, biological toxicity-based monitoring is necessary in order to make the effluent safer to the aquatic environment.

## References

[CR1] APHA-AWWA-WEF (2012) American Public Health Association/American Water Works Association/Water Environment Federation. Standard methods for the examination of water and wastewater 22^nd^ edn. APHA/AWWA/Water Environment Federation, Washington DC

[CR2] EPA US (2002). Methods for measuring the acute toxicity of effluents and receiving waters to freshwater and marine organisms.

[CR3] Hernando MD, Fernandez-Alba AR, Tauler R, Barcelo D (2005). Toxicity assays applied to wastewater treatment. Talanta.

[CR4] Libralato G, Ghirardini AV, Avezzù F (2010). Toxicity removal efficiency of decentralised sequencing batch reactor and ultra-filtration membrane bioreactors. Wat Res.

[CR5] Libralato G, Ghirardini AV, Francesco A (2010). How toxic is toxic? A proposal for wastewater toxicity hazard assessment. Ecotoxicol Environ Saf.

[CR6] Libralato G, Gentile E, Ghirardini AV (2016). Wastewater effects on Phaedodactylum tricornutum (Bohlin): setting up a clasification system. Ecol Indic.

[CR7] Madoni P (1994). A sludge biotic index (SBI) for the evaluation of the biological performance of activated sludge plants based on the microfauna analysis. Wat Res.

[CR8] Modern Water Microtox Acute Toxicity Overview, Modern Water N.D. (1998) http://www.coastalbio.com/images/Acute_Overview.pdf. Accessed 29 January 2015.

[CR9] Nalecz-Jawecki G, Baran S, Mankiewicz-Boczek J, Niemirycz E, Wolska L, Knapik J, Piekarska K, Bartosiewicz M, Pietowski G (2010). The first Polish interlaboratory comparison of the luminescent bacteria bioassay with three standard toxicants. Environ Prot Eng.

[CR10] Persoone G, Marsalek B, Blinova I, Törökne A, Zarina D, Manusadzianas L, Nalecz-Jawecki G, Tofan L, Stepanova N, Tothova L, Kolar B (2003). A practical and user-friendly toxicity classification system with microbiotests for natural waters and wastewaters. Environ Toxicol.

[CR11] Postma JF, De Valk S, Dubbeldam M, Maas JL, Tonkes M, Schipper CA, Kater BJ (2002). Confounding factors in bioassays with freshwater and marine organisms. Ecotoxicol Environ Saf.

[CR12] Ra JS, Kim HK, Chang NI, Kim SD (2007). Whole effluent toxicity (WET) tests on wastewater treatment plants with *Daphnia magna* and *Selenastrum capricornutum*. Environ Monit Assess.

[CR13] Swedish EPA (1997) Characterisation of discharges from chemical industry – The stork project. Swedish Environmental Protection Agency. Report no. 4766, Stockholm

[CR14] Tobajas M, Verdugo V, Polo AM, Rodriguez JJ, Mohedano AF (2015). Assessment of toxicity and biodegradability on activated sludge of priority and emerging pollutants. Environ Technol.

[CR15] Vasquez MI, Fatta-Kassinos D (2013). Is the evaluation of “traditional” physicochemical parameters sufficient to explain the potential toxicity of the treated wastewater at sewage treatment plants?. Environ Sci Pollut Res.

[CR16] Xiao Y, De Araujo C, Sze CC, Stuckey DC (2015). Toxicity measurement in biological wastewater treatment processes: a review. J Hazard Mater.

[CR17] Yi X, Kim E, Jo H-J, Schlenk D, Jung J (2009). A toxicity monitoring study on identification and reduction of toxicants from a wastewater treatment plant. Ecotoxicol Environ Saf.

